# PPFIA4 Promotes Colon Cancer Cell Proliferation and Migration by Enhancing Tumor Glycolysis

**DOI:** 10.3389/fonc.2021.653200

**Published:** 2021-05-20

**Authors:** Jia Huang, Meiling Yang, Zhaoxia Liu, Xiaoqian Li, Junfeng Wang, Nian Fu, Ting Cao, Xuefeng Yang

**Affiliations:** ^1^ Department of Gastroenterology, The Affiliated Nanhua Hospital, Hengyang Medical College, University of South China, Hengyang, China; ^2^ Department of Oncology, The Affiliated Nanhua Hospital, Hengyang Medical College, University of South China, Hengyang, China

**Keywords:** PPFIA4, colon cancer, glycolysis, PFKFB3/ENO2, invasion

## Abstract

Dysregulated glycolysis is one of the mechanisms employed by cancer cells to facilitate growth and metastasis. Here we aimed to characterize the PPFIA4 gene, as a glycolysis-related oncogene in promoting the proliferation and migration of colon cancer cells. Using bioinformatical tools including The Cancer Genome Atlas (TCGA) and Gene Expression Profiling Interactive Analysis (GEPIA), we found that PPFIA4 expression and methylation levels were higher in colon cancer tissues of different stages than in normal tissues. Higher PPFIA4 level was also positively correlated with poorer survival of patients. PPFIA4 upregulation also correlated with poor prognosis and higher clinical stages of colon cancer patients. Colon cancer cell viability, migration and migration were enhanced after PPFIA4 overexpression. EMT markers and glycolysis were upregulated after PPFIA4 overexpression. PPFIA4 expression was found to be positively correlated with PFKFB3 and ENO2 levels, while knockdown of PFKFB3 and ENO2 reduced cell proliferation, migration, invasion and glycolysis. PPFIA4 upregulation is a potential biomarker in colon cancer which promotes proliferation, migration, invasion and glycolysis. The upregulation of PFKFB3/ENO2 signaling by PPFIA4 is a potential mechanism underlying the oncogenic effects of PPFIA4.

## Introduction

Colon cancer is the fourth leading cause of cancer-related deaths worldwide, and new biomarkers for colon cancer are still urgently needed to development effective means to improve treatment outcomes. Emerging genomic, transcriptomic and proteomic studies on colon cancer have rapidly accumulated omics data that promise to bring novel biomarkers and drug targets to the clinic. Several established cancer databases and tools, which enable systemic analysis of clinical data on a large cohort of cancer patients. For instance, The Cancer Genome Atlas (TCGA) (1) and Gene Expression Profiling Interactive Analysis (GEPIA) (2) have tremendously facilitated the discovery of new genes aberrantly expressed in colon cancer, which have potential to become novel diagnostic and therapeutic targets, providing molecular basis for targeted therapies and advanced precision medicine for colon cancer treatment.

Among oncogenes identified in colon cancer, genes that regulate cancer cell metabolism have garnered growing interest as it is increasingly recognized that dysregulated cellular energetics metabolism is fundamentally connected to cancer proliferation, epigenetics and survival of patients (3). One of the key changes of metabolic pathways in cancer is the enhanced aerobic glycolysis, which is known as the Warburg effect (4). While normal cells generally adopt oxidative phosphorylation to transform glucose into carbonic anhydride, invasive cancer cells convert glucose to lactate, known as glycolysis, which only produces 2 ATP molecules (far less than the 26 ATP produced by complete oxidation of glucose) (5). While being apparently a waste of glucose, this strategy, coupled with enhanced glucose uptake by tumor cells, promotes the survival of cancers by making them insensitive to hypoxic conditions and increasing production of nucleosides and amino acids. In addition, lactate, the product of glycolysis, stimulates tumor invasion by enhancing migratory ability, immune escape, angiogenesis and radioresistance (6).

With the aim to identify new diagnostic and therapeutic biomarkers for colon cancer, we here performed a bioinformatics analysis and found that the PPFIA4 (protein tyrosine phosphatase receptor type F polypeptide interacting protein α-4) gene, which encodes for the liprin-alpha-4 protein (7), demonstrated significant differential expression in colon cancer. While being rarely studied, PPFIA4 has been reported in several studies to be potentially associated with aberrant metabolic processes (8–10), making it a gene of interest in cancer glycolysis. We validated that PPFIA4 was highly correlated with the survival and prognosis of colon cancer patients, and this study focused on evaluating the role of PPFIA4 in promoting proliferation, migration and invasion of colon cancer cells. More importantly, we showed that PPFIA4 was involved in the regulation of glycolysis, and silencing PPFIA4 attenuated glycolysis, suppressed cancer cell proliferation, migration and invasion. Mechanistically, we found that PPFIA4 could regulate the expression of glycolysis-related genes PFKFB3 and ENO2 (11). Findings of this study potentiated the use of PPFIA4 as a candidate diagnostic and therapeutic marker for colon cancer.

## Materials and Methods

### Bioinformatics Analysis

Data of patient survival were obtained and analyzed using the GEPIA database (Gene Expression Profiling Interactive Analysis, http://gepia.cancer-pku.cn/). TCGA analysis was performed on the UCLCN website (http://ualcan.path.uab.edu/).

### Cell Culture and Gene Transfection

All cell lines were acquired from American Type Culture Collection (ATCC) and maintained according to the recommendations of ATCC. All cells were passaged for less than two months and regularly tested for mycoplasma contamination. The PPFIA4 overexpression plasmid was constructed by inserting the PPFIA4 cDNA (Sinobiological, Beijing China) into pDNA-3.1 plasmid (Addgene, USA). Short-hairpin RNAs (shRNAs) for PPFIA4 knockdown (shRNA-1 and shRNA-2) were designed and synthesized by GenePharm (Shanghai, China). Small interfering RNAs (siRNAs) for PFKB3 and ENO2 were purchased from ThermoFisher Scientific (USA). A negative control (NC) siRNA serving as negative control was purchased from GenePharm (Cat#A06001). Transfection of the plasmids and oligonucleotides was conducted using Lipofectamine 2000 (ThermoFisher Scientific, Waltham, MA USA) using manufacturer’s recommendations.

### qRT-PCR

Total RNA extracted using the Trizol agent (ThermoFisher Scientific) was used in cDNA synthesis and real-time PCR was performed using the SYBR_PremixExTaq II kit (Takara, Dalian, China) and the CFX96 Real-Time PCR Detection System (Bio-Rad, Hercules, CA, USA). All primers were designed and synthesized by GenePharm.

### Cell Proliferation Assay

The proliferation of cells was analyzed by direct counting of cell numbers under a light microscope, using cell counting kit-8 (CCK-8) assay and colony formation assay. CCK-8 assay was conducted using reagents acquired from Sigma-Aldrich (USA). In colony formation assay, the number of colonies formed by cells plated in six-well plates at a density of 200 cells per well was counted at 2 weeks after seeding.

### Transwell Assays

Migration and invasion of cells were assessed in transwell assays using Transwell Cell Culture Inserts (BD Biosciences, USA) in accordance to manufacturer’s recommendations. In brief, cells were seeded on the top chamber at the density of 1×10^5^ cells per well in 100 μl serum-free medium) and the bottom chamber was filled with 800 µl medium containing 10% FBS. Migration assay was conducted in top chambers with no coating on the bottom, while invasion assay was performed in top chambers with Matrigel coating. After 36 h, cells migrated across the membrane were fixed using 4% PFA and stained with gentian violet. Cell numbers of five random fields of view were counted and the average number was reported.

### Western Blotting

The cells were lysed using RIPA buffer and protein content was measured with a BCA protein assay kit (Abcam), resolved by 10% SDS-PAGE gels and then electro-transferred onto nitrocellulose membranes. Then primary antibodies (Cell Signaling Technology, USA) were added to the membrane and incubated for 1 h at room temperature. The PFKFB3 (#13123) and ENO2 (#24330) were purchased from Cell Signaling Technology and diluted at 1:1000. Protein bands were visualized by electrochemiluminescence and protein expression was normalized using β-actin as a loading control.

### Detection of Levels of ATP, ECAR, Lactic Acid Production

The measurements of ATP, ECAR, and lactic acid production were performed using a previously reported protocol (12) using the Seahorse XFe96 Analyzer (Agilent Technologies, USA).

### Statistical Analysis

All data were presented as mean ± standard deviation (SD) and analyzed using Student’s t-tests, one- or two-way ANOVA followed by a *post hoc* test. Statistically significant results were determined using *P*< 0.05.

## Results

### PPFIA4 Correlates With Poor Survival of Colon Cancer Patients

**Figure 1:**
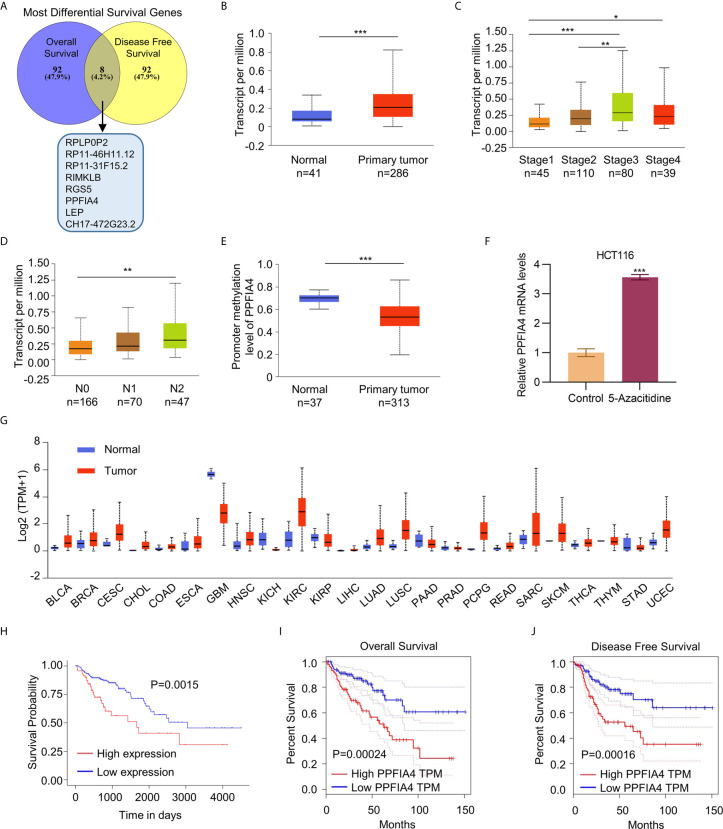
PPFIA4 correlates with poor survival outcomes in colon cancer. **(A)** Venn diagram shows the union set of Most Differential Survival Genes in colon cancer patients in GEPIA datasets. **(B)** Expression of PPFIA4 in normal and colon adenocarcinoma tissues in TCGA datasets. **(C)** Expression of PPFIA4 in colon adenocarcinoma patients with different tumor stages in TCGA datasets. **(D)** Expression of PPFIA4 in colon adenocarcinoma tumor tissues with different metastatic stages in TCGA datasets. **(E)** The methylation levels of PPFIA4 in normal and colon adenocarcinoma tumor tissues were analyzed in TCGA datasets. **(F)** The mRNA levels of PPFIA4 in HCT116 cells treated with 5-Azacytidine were determined by qPCR. **(G)** Kaplan–Meier plots of overall survival in colon adenocarcinoma patients stratified according to their PPFIA4 levels in TCGA datasets. **(H)** Expression of PPFIA4 across TCGA cancers with tumor and normal samples. **(I)** Analysis of overall survival of colon cancer patients using Kaplan–Meier plots. The patients were stratified according to their PPFIA4 levels in GEPIA datasets. **(J)** Analysis of disease-free survival in colon cancer patients using Kaplan–Meier plots. *p < 0.05, **p < 0.01, ***p < 0.001.

We first used the GEPIA dataset to screen for the top 100 most differential survival genes (MDSGs) in colon cancer, which significantly correlated with overall survival (OS) and disease-free survival (DFS) of patients (**Figure 1A**). Eight genes, including CH17-472G23.2, LEP, PPFIA4, RGS5, RIMKLB, RP11- 31F15.2, RP11-46H11.12, and RPLP0P2 were found to be associated with both OS and DFS. However, only LEP and PPFIA4 had protein expression analysis data in the database. After ruling out LEP as a potential biomarker using TCGA analysis (**Figures S1A–C**), we found that higher PPFIA4 levels were associated with primary tumor, higher stage (II-IV), and higher N stage (referring to a higher number of lymph node metastasis adjacent to tumor in the TNM staging system) (**Figures 1B–D**). In addition, the PPFI4 levels was higher in colon cancer tissues compare to the normal tissues (**Figure S1D**), and patients with lymph node metastasis showed higher PPIF4 levels compared to the patients without lymph node metastasis (**Figure S1E**). Higher promoter methylation levels of PPFIA4 were also found to be in the primary colon tumor than normal tissues (**Figure 1E**). We also validated that in HCT116 cells treated with 5-Azacitidine, PPFIA45 was upregulated (**Figure 1F**).

It was found that colon cancer patients with higher expression of PPFIA4 had poorer OS rate (p<0.05, **Figure 1G**). Then we confirmed that PPFIA4 overexpression was characteristic of many cancers (**Figure 1H**). We further analyzed the survival of PPFIA4 in colorectal cancer patients on the GEPIA website and found that colorectal cancer patients with higher expression of PPFIA4 showed poorer OS (p=0.00024) and recurrence-free survival (p=0.00016) (**Figures 1G–J** **)**.

### PPFIA4 Promotes Colon Cancer Cell Proliferation

**Figure 2:**
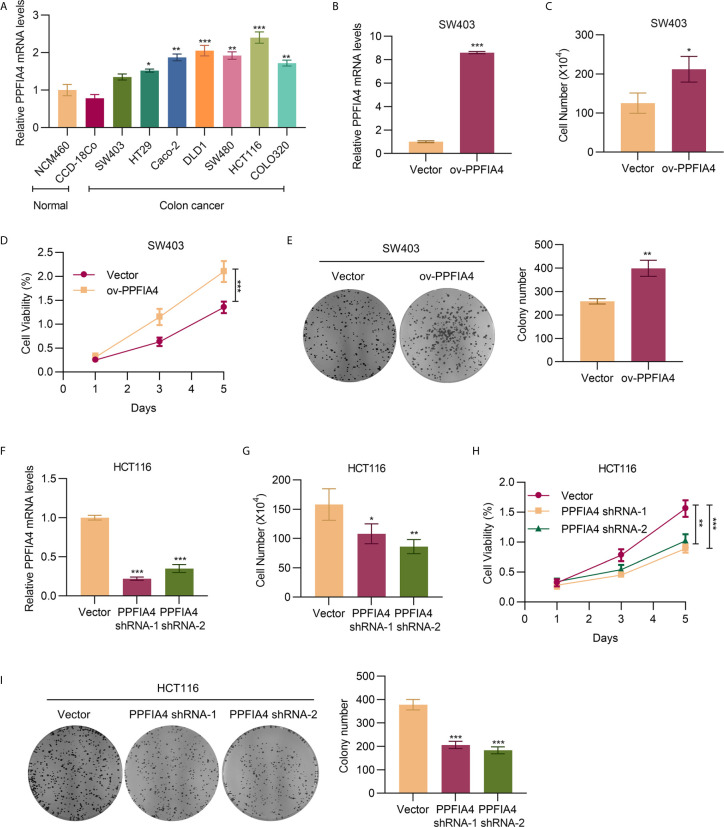
PPFIA4 promotes colon cancer cell proliferation. **(A)** The mRNA expression of PPFIA4 in colon cancer cell lines or normal colon cell lines. **(B)** qPCR analysis of the mRNA level of PPFIA4 in SW403 cells transfected with PPFIA4 expression plasmid. Viability of SW403 cells transfected with PPFIA4 expression plasmid determined using cell counting assay **(C)**, CCK8 assay **(D)**, and foci formation assay **(E)**. **(F)** The mRNA level of PPFIA4 in HCT116 cells transfected with PPFIA4 shRNA. Viability of HCT116 cells transfected with PPFIA4 shRNA determined by cell counting **(G)**, CCK8 assay **(H)** and foci formation assay **(I)**. *p < 0.05, **p < 0.01, ***p < 0.001.

We used qRT-PCR to detect the expression of PPFIA4 in colon cancer cell lines (**Figure 2A**), and found that the level of PPFIA4 was higher in colon cancer cells, including SW403, HT29, CAco-2, DLD1, SW480, HCT116, and COLO320, than that in normal colon cell lines, including NCM460 and CCD-18Co. Due to the lowest PPFIA4 expression in SW403 cells and the highest PPFIA4 expression among all colon cancer cell lines investigated in our study, we proceeded to overexpress PPFIA4 (ov-PPFIA4) in SW403 cells by transfecting PPFIA4 expression plasmid, as well as knocking down PPFIA4 using shRNAs in HCT116 cells. qRT-PCR analysis of PPFIA4 confirmed overexpression of PPFIA4 in SW403 cells (~8-fold upregulation compared to cells transfected with vector alone, p<0.001) (**Figure 2B**). We found that overexpression of PPFIA4 could promote the proliferation of SW403 cells as evaluated by cell counting (**Figure 2C**), CCK8 assay (**Figure 2D**) and colony formation assay (**Figure 2E**). Conversely, in HCT116 cells with PPFIA4 knockdown (as confirmed by qRT-PCR assay, **Figure 2F**), attenuated cell proliferation was observed in cell counting experiment (**Figure 2G**), CCK8 assay (**Figure 2H**) and colony formation assay (**Figure 2I**).

### PPFIA4 Promotes Colon Cancer Cell Migration and Invasion

**Figure 3:**
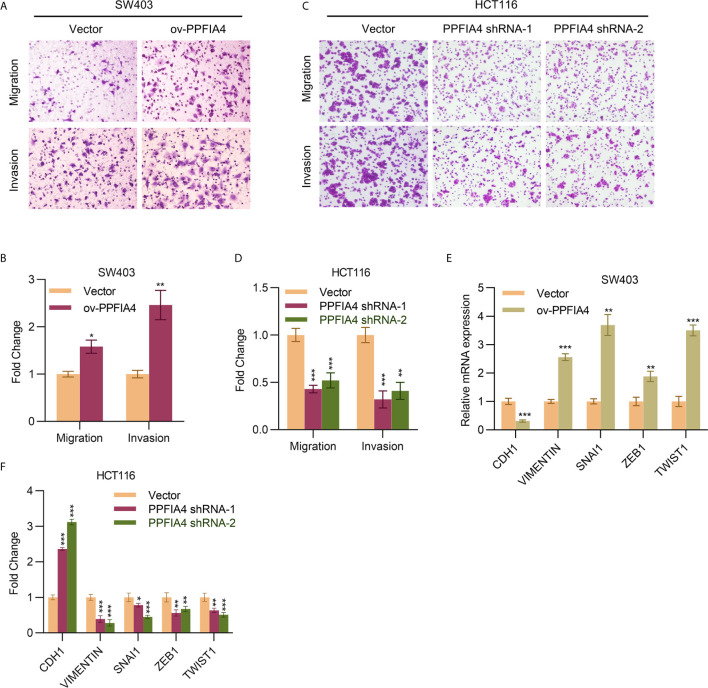
PPFIA4 induces colon cancer migration and invasion. **(A, B)** Transwell assay to determine cell migration and invasion of SW403 cells transfected with PPFIA4 expression plasmid **(A, B)** and HCT116 cells transfected with PPFIA4 shRNA **(C, D)**. **(E)** mRNA levels of EMT markers in SW403 cells transfected with PPFIA4 expression plasmid. **(F)** mRNA levels of EMT markers in HCT116 cells transfected with PPFIA4 shRNA. *p < 0.05, **p < 0.01, ***p < 0.001.

We next explored the role of PPFIA4 in colon cancer cell migration and invasion. We found that overexpression of PPFIA4 promoted the migration and invasion of SW403 cells (**Figures 3A, B**). On the other hand, knocking down PPFIA4 led to inhibition of migration and invasion of HCT116 cells (**Figures 3C, D**). All these data suggested a link between PPFIA4 level and the metastatic potential of tumors. Therefore, we analyzed markers of epithelial-mesenchymal-transition (EMT), which is one of the mechanisms that drive cancer metastasis, and we found that overexpression of PPFIA4 upregulated mesenchymal markers, including vimentin, SNAI1, ZEB1 and TWIST1 and downregulated the epithelial marker CDH1 in SW403 cells (**Figure 3E**). Conversely, knocking down PPFIA4 upregulated CDH1 and downregulated vimentin, SNAI1, ZEB1 and TWIST1 in HCT116 cells (**Figure 3F**).

### PPFIA4 Promotes Glycolysis

**Figure 4:**
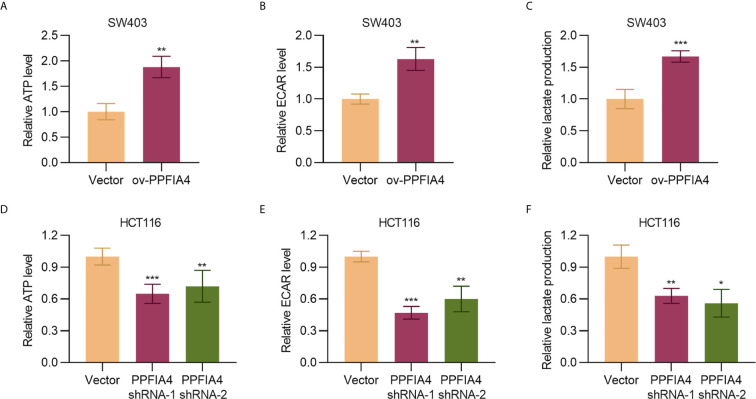
PPFIA4 promotes glycolysis. **(A)** Total ATP levels were measured in SW403 cells transfected with PPFIA4 expression plasmid or vector. **(B)** ECAR levels were measured in SW403 cells transfected with PPFIA4 expression plasmid or vector. **(C)** Extracellular lactate production in SW403 cells transfected with PPFIA4 expression plasmid or vector. **(D)** Total ATP levels were measured in HCT116 cells transfected with PPFIA4 expression plasmid or vector. **(E)** ECAR levels were measured in HCT116 cells transfected with PPFIA4 expression plasmid or vector. **(F)** Extracellular lactate production in HCT116 cells transfected with PPFIA4 expression plasmid or vector. *p < 0.05, **p < 0.01, ***p < 0.001.

As cancer is characteristic of dysregulated metabolism and cancer cells rely on glycolysis for enhanced energy production, we next evaluated the effect of PPFIA4 on colon cancer metabolism. Here we showed that overexpression of PPFIA4 significantly upregulated the levels of ATP (p<0.01) and ECAR, which detects the extracellular acid-producing capacity of cells and indirectly shows the glycolysis ability (p<0.01). The product of glycolysis, lactate (p<0.001), in SW403 cells was also increased (**Figures 4A–C**). Consistently, inhibiting PPFIA4 in HCT116 cells decreased the levels of ATP, ECAR and lactate production (**Figures 4D–F**). This indicated that PPFIA4 could promote the glycolysis process in colon cancer cells.

### PPFIA4 Regulates the Expression of PFKFB3/ENO2

**Figure 5:**
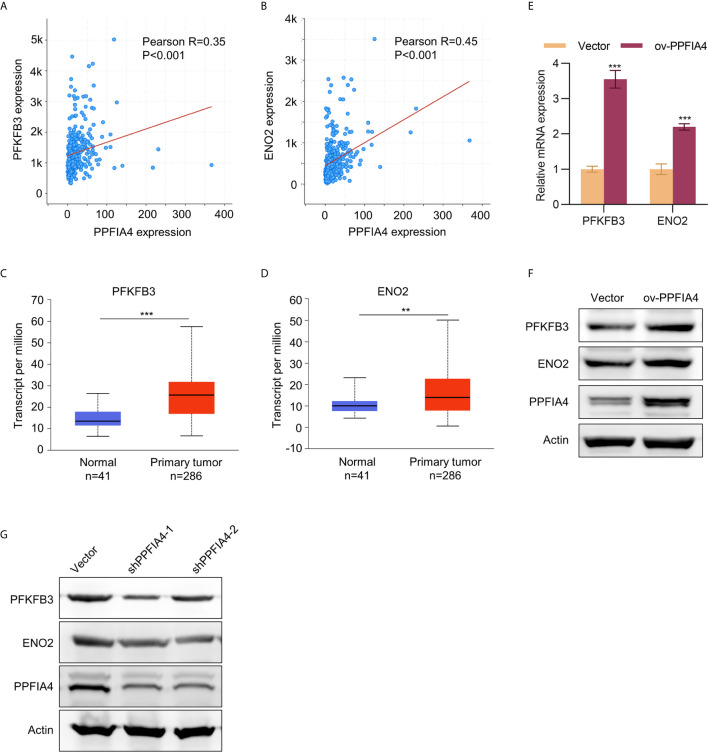
The expression of PFKFB3/ENO2 was regulated by PPFIA4. **(A)** The correlation between PPFIA4 and PFKFB3 was analyzed in TCGA colorectal adenocarcinoma dataset. **(B)** The correlation between PPFIA4 and ENO2 was analyzed in TCGA colorectal adenocarcinoma dataset. **(C)** Expression of PFKFB3 in normal and colon adenocarcinoma tissues in TCGA datasets. **(D)** Expression of ENO2 in normal and colon adenocarcinoma tissues in TCGA datasets. **(E, F)** The mRNA **(E)** and protein **(F)** expression of PFKFB3/ENO2 in SW403 or SW403-PPFIA4. **(G)** The protein levels of PFKFB3/ENO2 in HCT116 cells transfected with PPFIA4 shRNA. **p < 0.01, ***p < 0.001.

Next, we investigated the specific mechanism by which PPFIA4 promoted the proliferation, migration, invasion and glycolysis of colon cancer cells. We first analyzed the correlation between PPFIA4 and two glycolysis-related genes, ENO2 (enolase 2) and PFKFB3 (6-Phosphofructo-2-Kinase/Fructose-2,6-Biphosphatase 3), in the TCGA colon cancer database. We found that PPFIA4 showed significant positive correlations with expression levels of PFKFB3 and ENO2 (**Figures 5A, B**). Further, the levels of these three genes in the colon cancer database were analyzed in the UALCAN website, and the analysis showed that the levels of PFKFB3 and ENO2 were significantly higher in colon cancer tissues than in normal colon tissues (**Figures 5C, D**). The positive correlation between PPFIA4 and PFKFB3/ENO2 was then validated by qPCR (**Figure 5E**) and Western blot (**Figure 5F**) experiments, which showed that PPFIA4 overexpression in SW403 cells led to upregulated PFKFB3 and ENO2. On the other hand, knocking down PPFIA4 in HCT116 cells led to downregulation of PEKFB3 and ENO2 (**Figure 5G**).

### PPFIA4 Regulates PFKFB3 and ENO2 to Promote Colon Cancer Progression and Glycolysis

**Figure 6:**
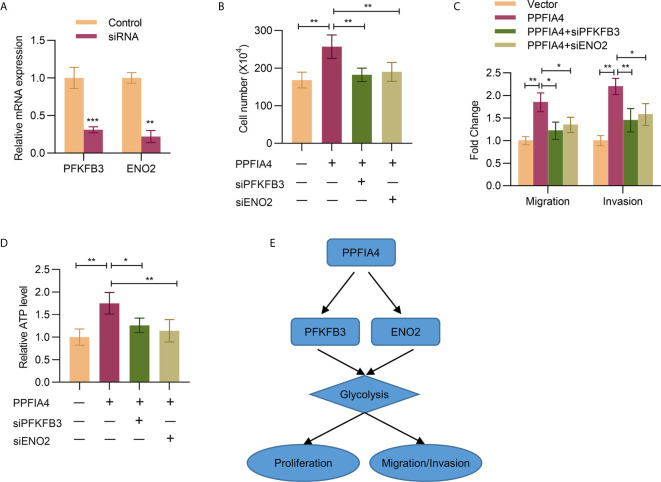
PPFIA4 regulates PFKFB3 and ENO2 to promote colon cancer progression and glycolysis. **(A)** The mRNA expression of PFKFB3 and ENO2 in SW403 cells transfected with PFKFB3 or ENO2 siRNA was determined by qPCR. **(B)** Cell viability of SW403 cells transfected with PPFIA4 expression plasmid and with or without PFKFB3/ENO2 siRNA was determined by cell count assay. **(C)** Transwell analysis of the migration and invasion of SW403 cells transfected with PPFIA4 expression plasmid and with or without PFKFB3/ENO2 siRNA **(D)** Total ATP levels were measured in SW403 cells transfected with PPFIA4 expression plasmid and with or without PFKFB3/ENO2 siRNA. **(E)** A diagram for the possible mechanisms. *p < 0.05, **p < 0.01, ***p < 0.001.

Finally, we wanted to verify whether knockdown of PFKFB3 or ENO2 would abrogate the tumor-promoting effects of PPFIA3. First, the knockdown efficiency of PFKFB3 or ENO2 by siRNAs was confirmed by qPCR experiments (**Figure 6A**). Then, we found that knocking down PFKFB3 or ENO2 indeed attenuated the effects of PPFIA4 overexpression on promoting cell proliferation (cell counting assay, **Figure 6B**), migration, invasion (transwell assay, **Figure 6C**) and glycolysis (measured by ATP levels, **Figure 6D**). A diagram that represents the possible mechanism of how PPFIA4 affects the glycolysis pathway, as well as cell proliferation and invasion, is shown in **Figure 6E**.

## Discussion

Our bioinformatics analysis identified PPFIA4 as a potential diagnostic and therapeutic biomarker in colon cancer. The PPFIA family includes PPFIA1-4, which possess different physiological functions in humans (7). PPFIA1 upregulation has been shown in 20% of breast cancers, and PPFIA1 was found to serve as an essential promoter in cancer cell migration and invasion (13). The involvement of PPFIA2 in the progression of myopia has also received considerable attention (14). Serum analysis of methylation of PPFIA3 genes has been suggested as an effective non-invasive detection method of gastric cancer (15). PPFIA4 is expressed in the human brain, heart and skeletal muscle in different degrees (7). Compared to other members of the PPFIA family, PPFIA4 has rarely been studied thus far, and our search for PPFIA4 on PubMed (16) has yielded 10 articles, only one of which is on PPFIA4 and cancer (17). No study has suggested a link between PPFIA4 and colon cancer. In our study, our TCGA analysis for the first time showed that PPFIA4 upregulation is a hallmark not limited to colon cancer, and a broad range of cancers are characterized by PPFIA4 upregulation (**Figure 1G**).

To validate the potential of PPFIA4 as a therapeutic target in colon cancer, our study focused on assessing the role of PPFIA4 in colon cancer cell proliferation, migration and invasion. We confirmed that all colon cancer cell lines showed upregulated PPFIA4 at different degrees. Notably, the SW403 cell line, which was previously shown to be relatively less aggressive and non-metastatic (18, 19), showed the lowest level of PPFIA4 upregulation among all colon cancer cell lines investigated in our study. Consistently, HCT116, which is a well-known aggressive colon cancer cell line (20), demonstrated the highest PPFIA4 expression. This finding is in agreement to the strong correlation between PPFIA4 level and cancer grade and patient survival (**Figure 1**), as PPFIA4 might be a valuable indicator of cancer cell aggressiveness. The correlation between PPFIA4 and phenotypes of colon cancer was also validated through ectopic overexpression of PPFIA4 in SW403 cells and silencing of PPFIA4 in HCT116 cells, which led to enhanced and suppressed cell proliferation, migration and invasion, respectively.

In our analysis using the GEPIA database, LEP is another gene identified other than PPFIA4. LEP is a gene that encodes leptin, which is an important regulator of adipose tissue mass and obesity (21). Consistent with the fact that obesity is a strong risk factor for cancer, leptin is also a known promoter of tumor growth in breast cancer (22). But in our study, LEP has not been found to be significantly associated with colon cancer stage. Other differentially expressed genes identified in our study included RPLPOP2, RP11-46H11.12, RP11-31F152, RIMKLB, RGS5, and CH17-472G23.2, which we did not analyze due to the lack of explicit description of their functions. But further studies are warranted to elucidate their roles in colon cancer.

Since currently available studies have implied the relationship between PPFIA4 and metabolism regulation (8–10), an important part of the present study was to evaluate if PPFIA4 upregulation was linked to glycolysis of colon cancer. Currently, a number of genes have been identified to be associated with glycolysis of cancers, such as ZBTB7A (23), HMGA1 (24) and CD36 (25). For example, Fang et al. reported that CD36 adopted a tumor-suppressing role by reprogramming glucose metabolism of tumors to repress colon cancer tumorigenesis (25). Despite these progresses, development of therapeutic targets to efficiently inhibit glycolysis of colon cancer is still an unachieved goal. Here we showed that overexpression and silencing of PPFIA4 resulted in increased and decreased glycolysis of colon cancer, respectively. The positive correlation between PPFIA4 and ENO2/PFKFB3 levels also corroborated that PPFIA4 was a potential regulator of glycolysis in colon cancer. To elucidate the molecular mechanism of PPFIA4 in regulating glycolysis, we explored if silencing of ENO2/PFKFB3 would abrogate the effects of PPFIA4 in promoting glycolysis. Our results confirmed that ENO2/PFKFB3 expression was indispensable in the regulation of glycolysis by PPFIA4.

Our data support the potential use of PPFIA4 silencing as another tool for reprogramming the energetic metabolism of colon cancer. However, our data are limited to *in vitro* studies and further *in vivo* studies are needed to confirm the efficacy of PPFIA4 silencing in cancer suppression and survival improvement in human tissues and/or animal models.

## Conclusion

Our study reports PPFIA4 as a potential novel therapeutic target in colon cancer. PPFIA4 expression is essential for colon cancer cell proliferation, migration, invasion and glycolysis. PPFIA4 silencing could be a strategy for reprogramming glycolysis of colon cancer, and its efficacy is dependent on ENO2/PFKFB3.

## Data Availability Statement

The raw data supporting the conclusions of this article will be made available by the authors, without undue reservation.

## Author Contributions

JH: Data curation, data analysis, drafting of the article, and final approval of the version to be published. MY: Data curation, data analysis, drafting of the article, and final approval of the version to be published. ZL: Data curation, data analysis, drafting of the article, and final approval of the version to be published. XL: Data curation, data analysis, drafting of the article, and final approval of the version to be published. JW: Data curation, data analysis, drafting of the article, and final approval of the version to be published. NF: Data curation, data analysis, drafting of the article, and final approval of the version to be published. TC: Study supervision, coordination, funding support, design of this study, drafting of the article, and final approval of the version to be published. XY: Study supervision, coordination, funding support, design of this study, drafting of the article, and final approval of the version to be published. All authors contributed to the article and approved the submitted version.

## Funding

The study was supported by the Novel Coronavirus Pneumonia Emergency Project of University of South China (2020-15 and 2020-25); the Hengyang Science and Technology Plan Project-Basic Research Project of Prevention and Treatment of the Novel Coronavirus Pneumonia (202010031577); the Scientific Research Project of Hunan Provincial Health and Family Planning Commission (A2017015); the Natural Science Foundation of Hunan Province, China (2016JJ5010); and the National Natural Science Foundation of China (81373465).

## Conflict of Interest

The authors declare that the research was conducted in the absence of any commercial or financial relationships that could be construed as a potential conflict of interest.
