# Supplementary material for: PPFIA4 Promotes Colon Cancer Cell Proliferation and Migration by Enhancing Tumor Glycolysis
Source: Front Oncol. 2021 May 20;11:653200. doi: 10.3389/fonc.2021.653200 (PMC8173052; doi:10.3389/fonc.2021.653200)
Supplement: Supplementary file 1 [file DataSheet_1.pdf]

Supplementary materials

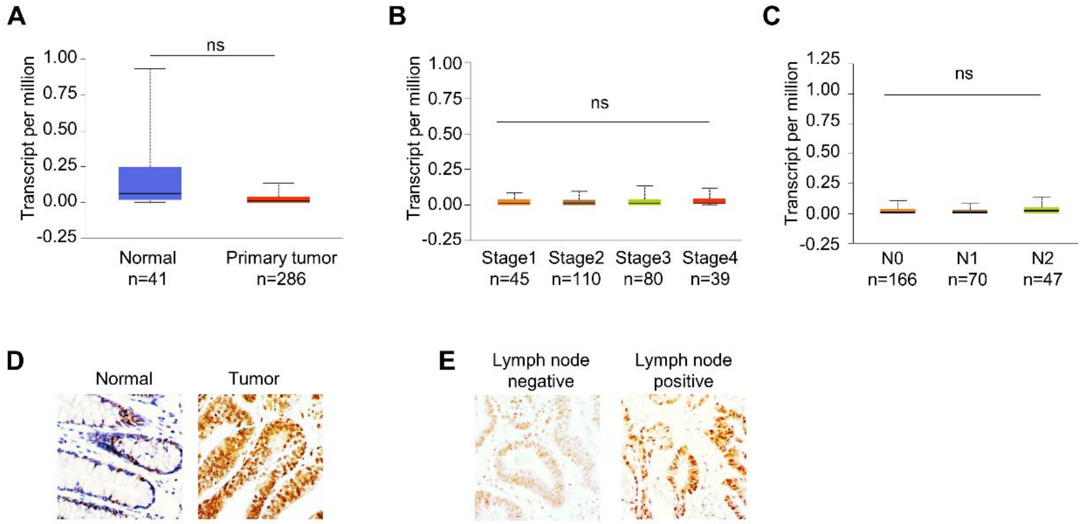

Figure S1. The clinical signature of LEP in colon cancer. (A) Expression levels of LEP in normal and colon adenocarcinoma tissues in TCGA datasets. (B) Expression levels of LEP in colon adenocarcinoma patients with different tumor stage in TCGA datasets. (C) Expression levels of LEP in colon adenocarcinoma tumor tissues with different metastatic stage in TCGA datasets. (D) IHC staining of PPFIA4 in colon cancer or normal tissues. (E) IHC staining of PPFIA4 in colon cancer patients with or without lymph node metastasis. Data are shown as mean ± S.D. ns, not significant.
